# Immune Responses in Discharged COVID-19 Patients With and Without Long COVID Symptoms

**DOI:** 10.1093/ofid/ofae137

**Published:** 2024-04-01

**Authors:** Yeming Wang, Li Guo, Dan Cui, Hui Zhang, Qiao Zhang, Lili Ren, Geng Wang, Xueyang Zhang, Tingxuan Huang, Lan Chen, Lixue Huang, Xinming Wang, Jinchuan Zhong, Ying Wang, Hui Li, Jianwei Wang, Bin Cao

**Affiliations:** Department of Pulmonary and Critical Care Medicine, Center of Respiratory Medicine, China-Japan Friendship Hospital, Institute of Respiratory Medicine, Chinese Academy of Medical Sciences, National Clinical Research Center for Respiratory Diseases, Beijing, China; National Health Commission Key Laboratory of Systems Biology of Pathogens and Christophe Mérieux Laboratory, Institute of Pathogen Biology, Chinese Academy of Medical Sciences & Peking Union Medical College, Beijing, China; Key Laboratory of Respiratory Disease Pathogenomics, Chinese Academy of Medical Sciences, Beijing, China; Department of Pulmonary and Critical Care Medicine, Center of Respiratory Medicine, China-Japan Friendship Hospital, Institute of Respiratory Medicine, Chinese Academy of Medical Sciences, National Clinical Research Center for Respiratory Diseases, Beijing, China; Department of Pulmonary and Critical Care Medicine, The 2nd Affiliated Hospital of Harbin Medical University, Harbin Medical University, Harbin, China; Department of Pulmonary and Critical Care Medicine, Center of Respiratory Medicine, China-Japan Friendship Hospital, Institute of Respiratory Medicine, Chinese Academy of Medical Sciences, National Clinical Research Center for Respiratory Diseases, Beijing, China; National Health Commission Key Laboratory of Systems Biology of Pathogens and Christophe Mérieux Laboratory, Institute of Pathogen Biology, Chinese Academy of Medical Sciences & Peking Union Medical College, Beijing, China; Key Laboratory of Respiratory Disease Pathogenomics, Chinese Academy of Medical Sciences, Beijing, China; National Health Commission Key Laboratory of Systems Biology of Pathogens and Christophe Mérieux Laboratory, Institute of Pathogen Biology, Chinese Academy of Medical Sciences & Peking Union Medical College, Beijing, China; Key Laboratory of Respiratory Disease Pathogenomics, Chinese Academy of Medical Sciences, Beijing, China; National Health Commission Key Laboratory of Systems Biology of Pathogens and Christophe Mérieux Laboratory, Institute of Pathogen Biology, Chinese Academy of Medical Sciences & Peking Union Medical College, Beijing, China; Key Laboratory of Respiratory Disease Pathogenomics, Chinese Academy of Medical Sciences, Beijing, China; Department of Pulmonary and Critical Care Medicine, WestChina Hospital, Sichuan University, Chengdu, China; School of Medicine, Tsinghua University, Beijing, China; National Health Commission Key Laboratory of Systems Biology of Pathogens and Christophe Mérieux Laboratory, Institute of Pathogen Biology, Chinese Academy of Medical Sciences & Peking Union Medical College, Beijing, China; Key Laboratory of Respiratory Disease Pathogenomics, Chinese Academy of Medical Sciences, Beijing, China; Department of Pulmonary and Critical Care Medicine, WestChina Hospital, Sichuan University, Chengdu, China; National Health Commission Key Laboratory of Systems Biology of Pathogens and Christophe Mérieux Laboratory, Institute of Pathogen Biology, Chinese Academy of Medical Sciences & Peking Union Medical College, Beijing, China; Key Laboratory of Respiratory Disease Pathogenomics, Chinese Academy of Medical Sciences, Beijing, China; Beijing Hospital, Beijing, China; National Health Commission Key Laboratory of Systems Biology of Pathogens and Christophe Mérieux Laboratory, Institute of Pathogen Biology, Chinese Academy of Medical Sciences & Peking Union Medical College, Beijing, China; Key Laboratory of Respiratory Disease Pathogenomics, Chinese Academy of Medical Sciences, Beijing, China; National Health Commission Key Laboratory of Systems Biology of Pathogens and Christophe Mérieux Laboratory, Institute of Pathogen Biology, Chinese Academy of Medical Sciences & Peking Union Medical College, Beijing, China; Key Laboratory of Respiratory Disease Pathogenomics, Chinese Academy of Medical Sciences, Beijing, China; National Health Commission Key Laboratory of Systems Biology of Pathogens and Christophe Mérieux Laboratory, Institute of Pathogen Biology, Chinese Academy of Medical Sciences & Peking Union Medical College, Beijing, China; Key Laboratory of Respiratory Disease Pathogenomics, Chinese Academy of Medical Sciences, Beijing, China; Department of Pulmonary and Critical Care Medicine, Center of Respiratory Medicine, China-Japan Friendship Hospital, Institute of Respiratory Medicine, Chinese Academy of Medical Sciences, National Clinical Research Center for Respiratory Diseases, Beijing, China; National Health Commission Key Laboratory of Systems Biology of Pathogens and Christophe Mérieux Laboratory, Institute of Pathogen Biology, Chinese Academy of Medical Sciences & Peking Union Medical College, Beijing, China; Key Laboratory of Respiratory Disease Pathogenomics, Chinese Academy of Medical Sciences, Beijing, China; Department of Pulmonary and Critical Care Medicine, Center of Respiratory Medicine, China-Japan Friendship Hospital, Institute of Respiratory Medicine, Chinese Academy of Medical Sciences, National Clinical Research Center for Respiratory Diseases, Beijing, China

**Keywords:** antibody, cellular response, COVID-19, immune, long COVID

## Abstract

The immune mechanisms of long coronavirus disease 2019 (COVID) are not yet fully understood. We aimed to investigate the severe acute respiratory syndrome coronavirus 2 (SARS-CoV-2)–specific memory immune responses in discharged COVID-19 patients with and without long COVID symptoms. In this cross-sectional study, we included 1041 hospitalized COVID-19 patients with the original virus strain in Wuhan (China) 12 months after initial infection. We simultaneously conducted a questionnaire survey and collected peripheral blood samples from the participants. Based on the presence or absence of long COVID symptoms during the follow-up period, we divided the patients into 2 groups: a long COVID group comprising 480 individuals and a convalescent group comprising 561 individuals. Both groups underwent virus-specific immunological analyses, including enzyme-linked immunosorbent assay, interferon-γ-enzyme-linked immune absorbent spot, and intracellular cytokine staining. At 12 months after infection, 98.5% (1026/1041) of the patients were found to be seropositive and 93.3% (70/75) had detectable SARS-CoV-2-specific memory T cells. The long COVID group had significantly higher levels of receptor binding domain (RBD)–immunoglobulin G (IgG) levels, presented as OD450 values, than the convalescent controls (0.40 ± 0.22 vs 0.37 ± 0.20; *P* = .022). The magnitude of SARS-CoV-2-specific T-cell responses did not differ significantly between groups, nor did the secretion function of the memory T cells. We did not observe a significant correlation between SARS-CoV-2-IgG and magnitude of memory T cells. This study revealed that long COVID patients had significantly higher levels of RBD-IgG antibodies when compared with convalescent controls. Nevertheless, we did not observe coordinated SARS-CoV-2-specific cellular immunity. As there may be multiple potential causes of long COVID, it is imperative to avoid adopting a “one-size-fits-all” approach to future treatment modalities.

A sample-representative cross-sectional survey showed that 7.3% of US adults reported long coronavirus disease 2019 (COVID-19) in 2022 [1]. Meanwhile, a meta-analysis that included 194 studies found that 45% of COVID-19 patients experienced long COVID, regardless of hospitalization status [2]. Similar post–acute infection syndromes have been observed in other viral infectious diseases, such as Ebola, Dengue, severe acute respiratory syndrome (SARS), and Chikungunya [3]. Despite this, the underlying pathogenic mechanisms that drive the development of these postacute sequelae remain unclear.

Numerous studies have revealed that COVID-19 patients, particularly severe cases, experience significant immune dysregulation during the acute phase of the infection [4–6]. Recent pathological studies have demonstrated that severe acute respiratory syndrome coronavirus 2 (SARS-CoV-2) RNA persists in multiple tissues of certain patients for prolonged periods, including the respiratory, cardiovascular, gastrointestinal, genitourinary, and central nervous systems, etc. [7–9]. Additionally, health care data from the US Department of Veterans Affairs have shown that treating patients with Paxlovid during the acute phase of SARS-CoV-2 infection can reduce the risk of developing long COVID by 26% [10]. Our previous research has indicated that most recovered patients 12 months after moderate to critical infection can develop sustained humoral and cellular immunity [11]. However, there is currently no conclusive evidence to suggest that viral fragments can form a reservoir that triggers sustained immune perturbation in the host, leading to the development of long COVID. While several studies have emerged to explore the potential immune dysregulation and immune memory of long COVID, the results have been inconsistent [12–16].

In light of the profound global impact of COVID-19, we aimed to investigate the mechanisms underlying long COVID by comparing the humoral and cellular immune profiles of patients with long COVID with those who had fully recovered.

## METHODS

### Study Design and Participants

This study was done at Jin Yin-tan Hospital, the first designated hospital for patients with COVID-19 in Wuhan, Hubei, China. We included all patients with laboratory-confirmed COVID-19 who were discharged from Jin Yin-tan Hospital between January 7, 2020, and May 29, 2020 (see full inclusion and exclusion criteria in the Supplementary Data, page 1) [17]. To minimize the potential impact of host immune status, this study further excluded individuals with (1) a history of malignant tumors, (2) rheumatic immune system disease history, or (3) long-term glucocorticoid administration. Illness severity was defined according to the 7-scale disease severity of COVID-19 patients [17].

**Figure 1.**
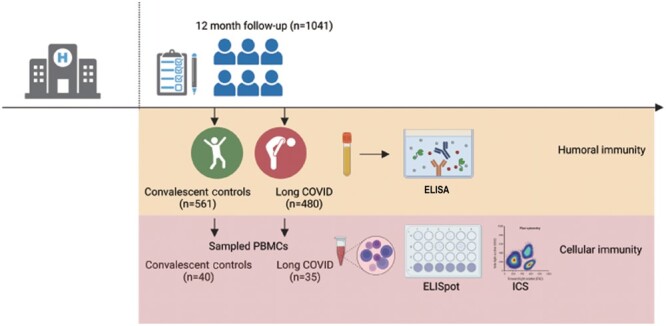
Flowchart of the study. Created with BioRender.com. Abbreviations: COVID, coronavirus disease 2019; ELISA, enzyme-linked immunosorbent assay; ELISpot, enzyme-linked immune absorbent spot; ICS, intracellular cytokine staining; PBMCs, peripheral blood mononuclear cells.

This follow-up study was performed at the Jin Yin-tan Hospital research center between December 16, 2020, and January 27, 2021, which was nearly 12 months after infection. Questionnaires and peripheral blood were collected simultaneously. Patients were divided into 2 groups based on the presence or absence of sequela symptoms during the follow-up period: the long COVID (LC) group and the convalescent controls (CC) group, respectively. The LC group was defined as patients who, at 12 months following their initial SARS-CoV-2 infection, reported at least 1 persistent symptom commonly associated with long COVID. This definition was predated but aligns with the World Health Organization Delphi consensus criteria [18]. All participants were infected with the original strain and had not received SARS-CoV-2 vaccination at the time of blood collection. No reinfection records were available. Figure 1 provides an overview of the study design.

The study was approved by the Ethics Committee of Institutional Review Boards of the Wuhan Research Center for Communicable Disease Diagnosis and Treatment, Chinese Academy of Medical Sciences (KY-2020–80.01).

### Immunological Evaluation

The whole blood collected from the 1041 participants was processed into plasma and peripheral blood mononuclear cells (PBMCs) within 12 hours, as described previously [11]. Due to constraints in our testing capacity and the limited availability of blood samples, we were not able to perform T-cell response tests on all study participants. Among the participants, 75 had adequate PBMCs for assessing SARS-CoV-2-specific memory T cells.

Plasma samples obtained from 1041 participants were subjected to enzyme-linked immunosorbent assay to measure the absorbance value at 450 nm (OD450) for immunoglobulin (Ig)M, IgA, and IgG antibodies against RBD, spike, nucleoprotein (N) protein. The cutoff value for seropositive was calculated by adding 3 SDs to the mean OD450 value of negative plasma antibodies, resulting in the following cutoff values: N-IgM = 0.30; S-IgM = 0.24; RBD-IgM = 0.20; N-IgA = 0.20; S-IgA = 0.26; RBD-IgA = 0.20; IgG = 0.20; RBD-IgG = 0.20. Sera from 135 randomly selected participants were titrated on Vero cells using a microneutralization assay to detect the presence of neutralizing antibodies against the original Wuhan SARS-CoV-2 strain.

We evaluated the SARS-CoV-2-specific memory T-cell responses in PBMCs obtained from 75 participants by measuring the interferon-γ-enzyme-linked immune absorbent spot (IFN-γ-ELISpot). The PBMCs were stimulated with 4 SARS-CoV-2 peptide pools containing 347 15- to 18-mer peptides that overlap by 10 amino acid residues and span the spike, N, membrane (M), envelope (E), open reading frame (ORF) 3a, ORF6, ORF7a, and ORF8 proteins of SARS-CoV-2. The spike pool contained 177 peptides; the NP pool contained 59 peptides; the M pool contained 29 peptides; and the E/ORF pool contained 82 peptides (E = 9, ORF3a = 35, ORF6 = 7, ORF7a = 15, ORF8 = 16, respectively). The number of immunospots in the stimulated PBMCs represented the magnitude of the immune response of SARS-CoV-2-specific memory T cells.

Intracellular cytokine staining (ICS) was employed to evaluate the activation and secretion of SARS-CoV-2-specific memory CD4+ and CD8+ T cells. Upon stimulation with the SARS-CoV-2 peptide pools, memory T cells were activated and produced cytokines including IL-2, IFN-γ, and tumor necrosis factor (TNF)–α. The cytokine-producing CD4+ and CD8+ T cells were sorted and counted using flow cytometry. To obtain the SARS-CoV-2-specific T-cell response, the activation number of the background control well was subtracted from the detection well before further analysis.

A detailed description of the immunological evaluation methods is available in the Supplementary Data, pages 1–3.

### Statistical Analysis

Continuous variables were described using mean (SD) or median (interquartile range [IQR]), while categorical variables were presented as frequency (proportion, %). A comparative analysis between the LC and CC groups was performed using the independent-samples *t* test, chi-square test, or Mann-Whitney *U* test, as appropriate. To determine the independent predictor of plasma RBD-IgG levels, we performed a multiple linear regression analysis with demographic variables (age and gender) as covariates. A Spearman correlation analysis was used to examine the correlation between antibody and ELISpot results. Statistical analysis and graphing were conducted using GraphPad Prism 8 software. A 2-sided *P* value of <.05 was considered statistically significant.

## RESULTS

### Clinical Characteristics of the Participants

In this study, 1041 discharged COVID-19 patients were eligible for analysis. Their mean age (SD) was 56.7 (12.5) years. Of these patients, 46.1% were women, and 40.1% had comorbidities. The duration from symptom onset to follow-up (IQR) was 349.0 (337.0–361.0) days. For the acute phase of COVID-19, defined as the period during hospitalization, 26.2% of patients were categorized as severity scale 3, 67.4% as scale 4, and 6.5% as scales 5–6. All patients were infected with the original SARS-CoV-2 strain during the first wave of the pandemic, and none had received the SARS-CoV-2 vaccine before the study.

Of the 1041 patients, 480 reported persistent sequelae symptoms (LC group), while 561 patients fully recovered without symptoms (CC group). Among the LC group patients, 233 individuals (48.5%) reported 1 symptom, while 247 individuals (51.5%) reported 2 or more symptoms. The most frequently reported symptoms in the LC group were fatigue/muscle weakness (34.4%), joint pain (29.0%), and sleep disorders (21.3%) during the 12-month follow-up (Supplementary Figure 1). More women (50% vs 42.8%; *P* = .020) and patients with cardiovascular diseases (9.4% vs 4.0%; *P* = .001) were in the LC group compared with the CC group. For more detailed baseline clinical characteristics of the participants, see Supplementary Table 1.

### SARS-CoV-2-Specific Antibody Levels Between LC and CC Patients

Plasma samples were collected from 1041 COVID-19 patients and tested for the presence of IgM, IgA, and IgG antibodies against RBD, spike, and N proteins. Twelve months postinfection, 98.5% (1026) of the patients were found to be seropositive (at least 1 specific antibody against RBD, spike, or N protein). The majority of patients had seropositive IgG, while the proportion of seropositive IgM and IgA was relatively low, suggesting that the patients had entered the recovery phase (Table 1).

**Table 1.** Seropositivity of COVID-19 Patients During 12-Month Follow-up

…	IgM	IgA	IgG
RBD	2.8% (29/1041)	2.4% (25/1041)	94.3% (982/1041)
S	2.6% (27/1041)	4.6% (48/1041)	95.2% (991/1041)
N	1.0% (10/1041)	3.4% (35/1041)	69.8% (727/1041)

Seropositivity was tested by ELISA. Cutoff values of OD450 were determined by calculating the mean absorbance at 450 nm of negative plasma plus 3-fold SD values.

Abbreviations: COVID-19, coronavirus disease 2019; ELISA, enzyme-linked immunosorbent assay; Ig, immunoglobulin; N, nucleoprotein; RBD, receptor binding domain; S, spike.

**Figure 2.**
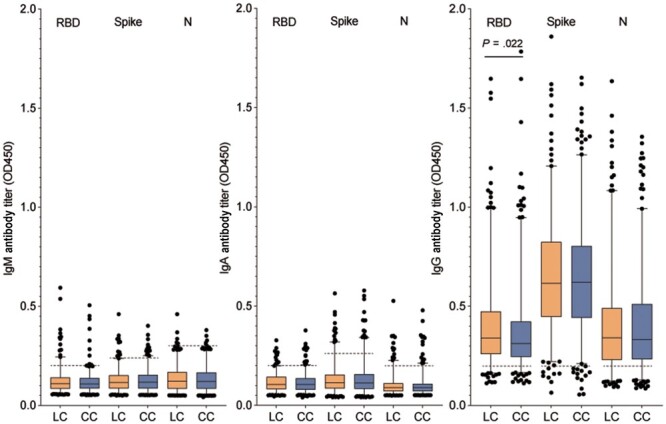
SARS-CoV-2 specific antibodies (OD450) of LC and CC patients. RBD-, spike-, and N-IgM, IgA, IgG antibody (OD450) between long COVID patients and convalescent controls during 12-month follow-up. Dashed line = the positive cutoff value for the serum antibodies. Abbreviations: CC, convalescent controls; COVID, coronavirus disease 2019; LC, long COVID; N, nucleoprotein; RBD, receptor binding domain; SARS-CoV-2, severe acute respiratory syndrome coronavirus 2.

To further explore the potential link between long COVID and humoral immune response, we compared SARS-CoV-2-specific antibody levels presented as OD450 values between LC patients and CC patients. We found that the RBD-IgG (OD450) in LC patients was significantly higher than that in CC patients (0.40 ± 0.22 vs 0.37 ± 0.20; *P* = .022). However, no statistical difference was observed in other antibodies between the LC and CC groups (Figure 2). To fully account for the impact of demographic variables on RBD-specific IgG antibody levels in the LC and CC groups, we conducted a multiple linear regression analysis. Our statistical models, which included the age and gender of the participants, indicated that long COVID status was a significant and positive predictor of anti-RBD humoral response (Supplementary Figure 2). These results suggest that SARS-CoV-2-specific humoral immune responses are maintained in LC patients.

We randomly selected 135 COVID-19 patients for microneutralization assays, with 71 in the LC group and 64 in the CC group. The results revealed that 80.7% of the patients (109 out of 135) exhibited neutralizing activity against the original strain of SARS-CoV-2 12 months after infection, with similar proportions in the LC and CC groups (83.1% and 78.1%, respectively). Furthermore, we found no significant difference in 50% neutralization titer (NT50) between the 2 groups (31.93 ± 35.83 vs 37.79 ± 45.67; *P* > .05) (Supplementary Figure 3).

### Ex Vivo Assessment of Memory T-Cell Responses Specific to SARS-CoV-2 Between LC and CC Patients

Out of the total number of participants, 75 had adequate PBMCs for assessing SARS-CoV-2-specific memory T cells, with 35 in the LC group and 40 in the CC group. The baseline clinical characteristics of all 75 participants are presented in Supplementary Table 2.

**Figure 3.**
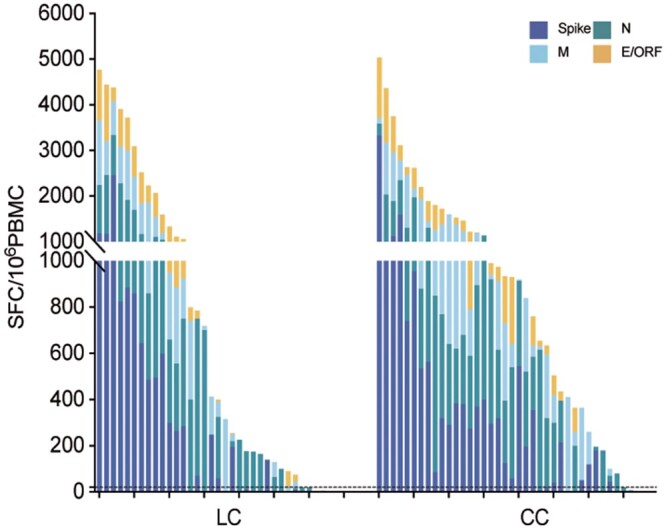
Magnitude of SARS-CoV-2-specific memory T cells of COVID-19 patients by IFN-γ-ELISpot. PBMCs from 75 COVID-19 patients were stimulated with the SARS-CoV-2 peptide pools, and the production of IFN-γ was detected by ELISpot. Each bar in the graph represents the overall T-cell response of an individual to the tested peptide pools, with different colors indicating the response of T cells specific to different viral proteins. The dashed line represents the positive cutoff value, namely magnitude ≥20 s.f.u./10^6^ PBMCs. Abbreviations: COVID-19, coronavirus disease 2019; E, envelope protein; ELISpot, enzyme-linked immunospot assay; IFN-γ, interferon-γ; PBMC, peripheral blood mononuclear cell; SARS-CoV-2, severe acute respiratory syndrome coronavirus 2.

After incubation of PBMCs with 4 peptide pools (containing 347 overlapping peptides of SARS-CoV-2), we detected the memory T cells using the ex vivo IFN-γ-ELISpot. We found that 70 of the 75 hospitalized COVID-19 patients (93.3%) had detectable SARS-CoV-2-specific memory T cells (responded to at least 1 SARS-CoV-2 peptide pool) 12 months after infection. These results suggest that individuals who have recovered from COVID-19 possess long-lasting SARS-CoV-2-specific memory T cells. But the magnitude of T-cell response displays significant heterogeneity (Figure 3).

**Figure 4.**
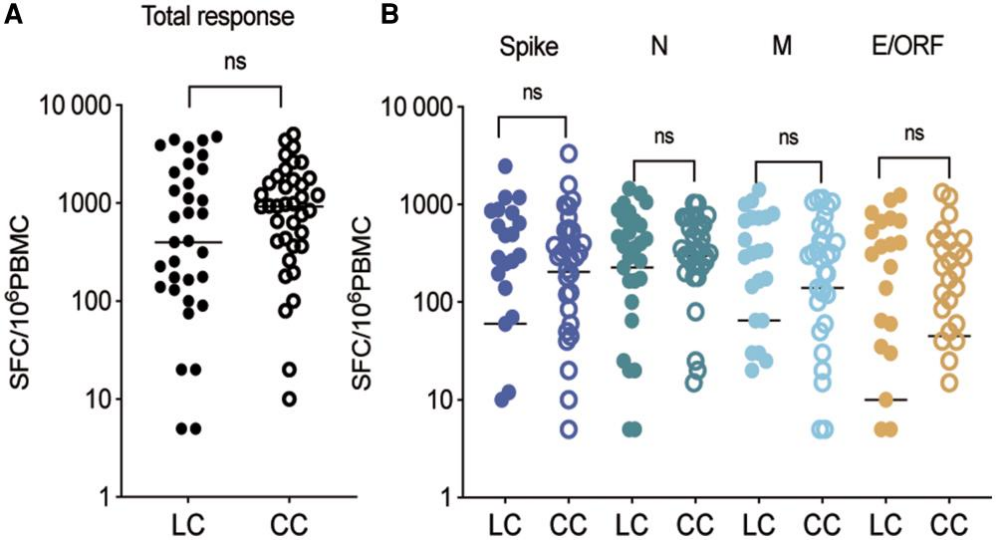
Comparison of SARS-CoV-2-specific memory T-cell magnitude in LC and CC patients. *A*, Total magnitude of T cells specific to the SARS-CoV-2 peptide pools in LC and CC patients, calculated as the sum of responses against spike, N, M, and E/ORF proteins. *B*, Comparison of magnitude of SARS-CoV-2-specific T cells against spike, N, M, and E/ORF proteins in the LC group and CC group, respectively. The short line represents the median. Abbreviations: CC, convalescent control; COVID, coronavirus disease 2019; E, envelope protein; ELISA, enzyme-linked immunosorbent assay; Ig, immunoglobulin; LC, long COVID; N, nucleoprotein; ORF, open reading frame; RBD, receptor binding domain; S, spike; SARS-CoV-2, severe acute respiratory syndrome coronavirus 2.

We further investigated the variation in the magnitude of SARS-CoV-2-specific T-cell responses between LC patients and those in the CC group. The analysis revealed that there was no significant difference in the mean number of virus-specific T-cell response immunospots between the 2 groups (1179.46 ± 1500.43 vs 1196.63 ± 1223.39; *P* = .957). Furthermore, when stratified according to the SARS-CoV-2 virus protein, there was no significant difference in the magnitude of virus-specific T-cell responses to spike, N, M, and E/ORF proteins between the LC and CC groups (Figure 4).

### Assessing Functional Activity of SARS-CoV-2-Specific Memory CD4+ and CD8+ T Cells in LC and CC Patients

To further explore the role of T-cell activation and secretion function in the development of long COVID, we used ICS and flow cytometry to sort and count cytokine production, including IL-2, IFN-γ, and TNF-α, in PBMCs from both LC and CC patients after SARS-CoV-2 peptide stimulation.

Among all participants, the proportions of total cytokine-producing CD4+ and CD8+ T cells in response to spike protein were 2.32% and 1.81%, respectively (Supplementary figure 5). We compared the abundance of cytokine-producing CD4+ and CD8+ T cells in the LC and CC patient groups and found no statistically significant differences.

**Figure 5.**
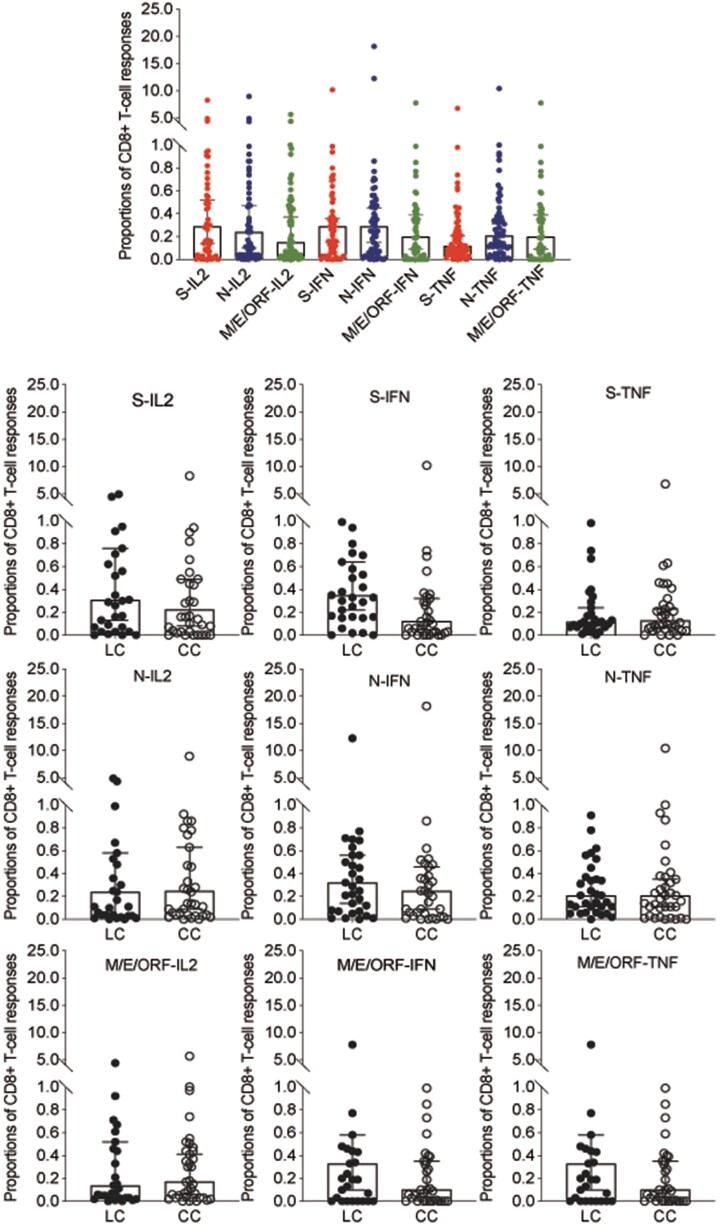
Secretory function of memory CD8+ T cells in response to SARS-CoV-2 protein. The top image, Proportions of SARS-CoV-2-specific CD8+ T cells producing IL-2, IFN-γ, and TNF-α in response to spike, N, and M/E/ORF stimulation in PBMCs. The bottom images, Proportions of CD8+ T cells producing IL-2, IFN-γ, and TNF-α in response to stimulation with spike, N, and M/E/ORF between the LC and CC groups. Filled circles represent LC patients; open circles represent CC patients. “S-IL2” stands for S-protein stimulated IL-2+ CD8+ T cells; “N-IL2” stands for N-protein stimulated IL-2+ CD8+ T cells; “M/E/ORF-IL2” stands for M/E/ORF-protein stimulated IL-2+ CD8+ T cells, and so on. Abbreviations: CC, convalescent control; COVID, coronavirus disease 2019; E, envelope protein; ELISA, enzyme-linked immunosorbent assay; IFN, interferon; IL, interleukin; LC, long COVID; M, membrane; N, nucleoprotein; ORF, open reading frame; PBMCs, peripheral blood mononuclear cells; RBD, receptor binding domain; S, spike; SARS-CoV-2, severe acute respiratory syndrome coronavirus 2; TNF, tumor necrosis factor.

**Figure 6.**
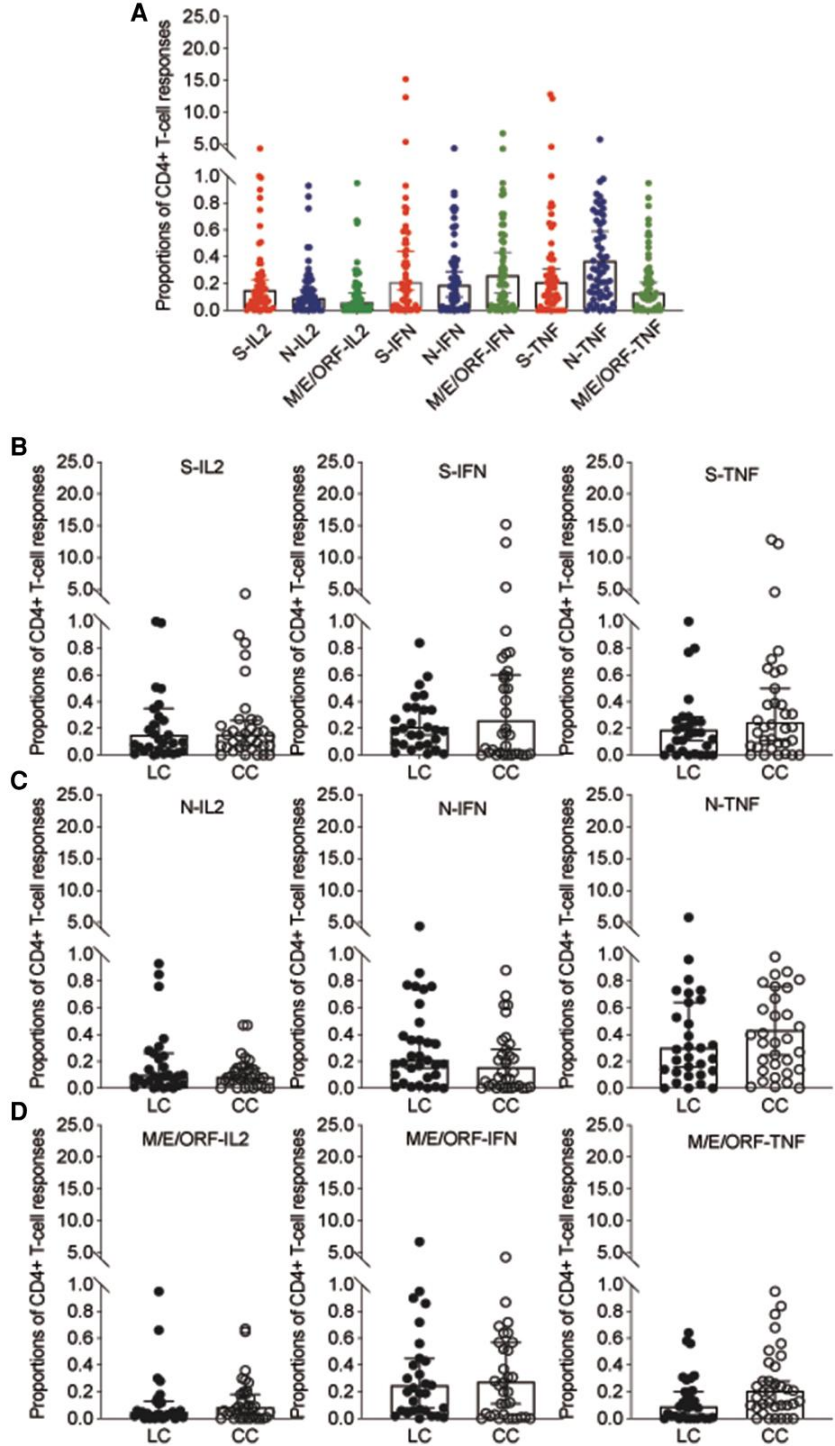
Secretory function of memory CD4+ T cells in response to SARS-CoV-2 protein. *A*, Proportions of SARS-CoV-2-specific CD4+ T cells producing IL-2, IFN-γ, and TNF-α in response to spike, N, and M/E/ORF stimulation in PBMCs. *B–D*, Proportions of CD8+ T cells producing IL-2, IFN-γ, and TNF-α in response to stimulation with spike (*B*), N (*C*), and M/E/ORF (*D*) between the LC and CC groups. Filled circles represent LC patients; open circles represent CC patients. “S-IL2” stands for S-protein stimulated IL-2+ CD4+ T cells; “N-IL2” stands for N-protein stimulated IL-2+ CD4+ T cells; “M/E/ORF-IL2” stands for M/E/ORF-protein stimulated IL-2+ CD4+ T cells, and so on. Abbreviations: CC, convalescent control; COVID, coronavirus disease 2019; E, envelope protein; ELISA, enzyme-linked immunosorbent assay; IFN, interferon; IL, interleukin; LC, long COVID; M, membrane; N, nucleoprotein; ORF, open reading frame; PBMCs, peripheral blood mononuclear cells; RBD, receptor binding domain; S, spike; SARS-CoV-2, severe acute respiratory syndrome coronavirus 2; TNF, tumor necrosis factor.

Furthermore, the proportions of SARS-CoV-2-specific CD8+ T cells producing IL-2, IFN-γ, and TNF-α in response to spike, N, and M/E/ORF stimulation in PBMCs are shown in Figure 5. No significant differences were observed when comparing these proportions between the LC and CC groups. Similarly, we also assessed the proportions of virus-specific CD4+ T cells producing cytokines in response to spike, N, or M/E/ORF protein stimulation and found no significant differences between the groups (Figure 6).

### Correlation Between SARS-CoV-2-Specific Memory T-Cell Responses and Antibodies

**Figure 7.**
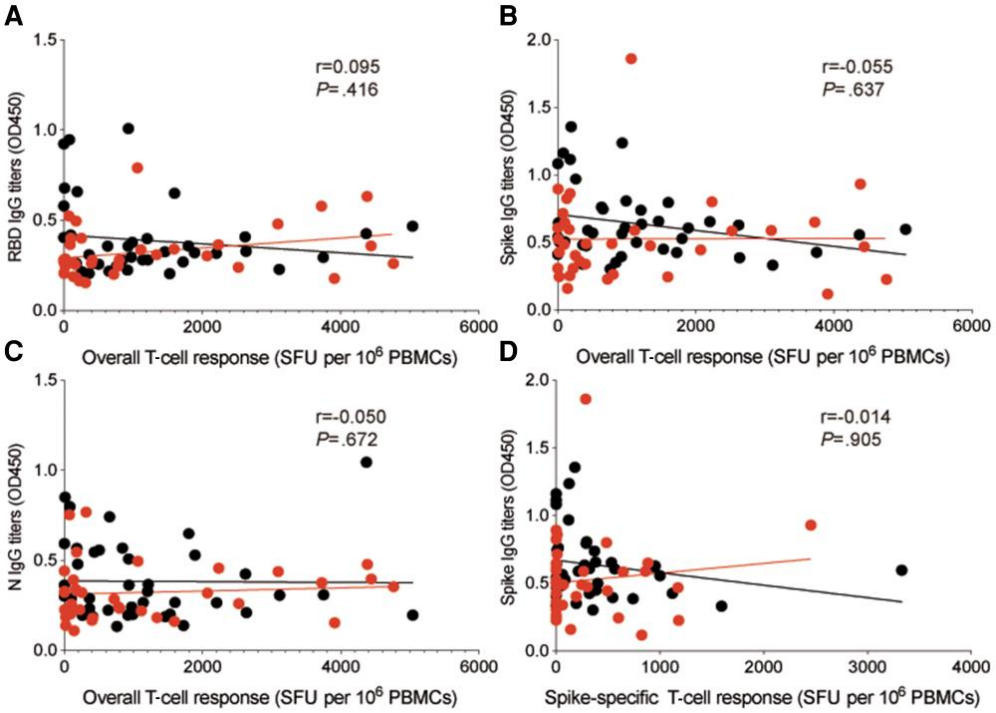
Correlation between SARS-CoV-2-specific memory T-cell responses and IgG antibody (OD450). The figure displays the correlation between RBD- (*A*), spike- (*B*), and N- (*C*) specific IgG antibodies (OD450) and magnitude of overall virus-specific T-cell responses. *D*, Correlation between spike-specific IgG antibodies (OD450) and magnitude of spike-specific T-cell responses. Red dots represent LC patients; black dots represent CC group patients. Abbreviations: CC, convalescent control; COVID, coronavirus disease 2019; Ig, immunoglobulin; LC, long COVID; N, nucleoprotein; RBD, receptor binding domain; SARS-CoV-2, severe acute respiratory syndrome coronavirus 2.

To investigate the potential relationship between SARS-CoV-2-specific memory T-cell responses and antibodies, we examined the correlation between the magnitude of ELISpot results and RBD-, spike-, N-, and M-specific IgG antibodies (OD450). However, we did not observe any significant correlation between them, as shown in Figure 7. Additionally, we found no significant correlation between the magnitude of spike-specific memory T cells and spike-specific IgG (OD450). We also performed separate analyses for the LC and CC patient groups, but no correlation was significant in either group. Although not statistically significant, it is noteworthy that the correlation trends among LC and CC patients were inconsistent.

## DISCUSSION

In this study, we evaluated the humoral and cellular immune responses specific to SARS-CoV-2 in a cohort of 1041 hospitalized COVID-19 patients at 12 months after infection. Consistent with previous studies, the majority of patients developed durable adaptive immune memory. Notably, patients with long COVID exhibited higher levels of RBD-IgG antibodies than convalescent controls. However, we did not detect any significant differences in the magnitude or secretion function of the memory T cells between the 2 groups. Additionally, there is no correlation between cellular and humoral immunity in the LC group.

Our study with a large sample size confirmed that long COVID patients have significantly higher SARS-CoV-2-specific IgG antibody levels compared with those who have fully recovered at 12 months after infection. These findings have also been reported in previous studies with relatively smaller sample sizes [12, 16]. Additionally, Jacob et al. [14] found that the avidity of spike-IgG antibodies in long COVID patients was sustained at a higher level over time, in contrast to the gradual decay observed in recovered patients. Although viral fragments may not be detected in the upper respiratory tract, previous research has indicated that the failure to detect viral fragments does not rule out the possibility of persistent viral fragments in vivo [19, 20]. Additionally, factors such as autoimmunity, potential re-exposure to the virus, and chronic inflammation could contribute to the sustained high levels of RBD-IgG antibodies as they indicate a persistently active immune system. These mechanisms may play a role in the continuation of long COVID symptoms.

But after examining the SARS-CoV-2-specific memory T cells in both LC and CC patients, we found no significant differences in response magnitude and cytokine secretion function between the 2 groups. These findings are consistent with the results of Fang et al. [15] in 33 COVID-19 patients. However, some studies have shown impaired T-cell function in long COVID patients. Turner et al. [21] reported that impaired IFN-γ secretion was observed in circulating CD8+ T cells in a patient with persistently positive SARS-CoV-2. Peluso and colleagues [13] also found a decrease in CD8+ T cells responsible for viral clearance in patients with long COVID 4 months after infection, while there was preferential activation of the CD8+ T cells that cause lung tissue damage. The relationship between SARS-CoV-2-specific memory T cells and the occurrence and persistence of long COVID remains unclear and requires further investigation. However, it should be noted that our research on SARS-CoV-2-specific cellular immunity was conducted with a relatively small sample size, and therefore, the conclusions drawn from it should be interpreted with caution.

This study reveals that the humoral and cellular aspects of SARS-CoV-2-specific immunity in long COVID patients are not well coordinated. Unlike memory T cells, memory B cells that secrete SARS-CoV-2-specific IgG antibodies in patients continue to increase in number for months after infection [22–25]. However, circulating antibodies may not accurately reflect the richness and persistence of the host's immune memory against SARS-CoV-2, as observed by Dan et al. [22]. Nevertheless, there is still much unknown regarding the adaptive immune response in different long COVID patients, such as whether the immune response is overactivated or overinhibited and whether the response level is high or low. Further investigation is necessary to elucidate the interplay between SARS-CoV-2-specific memory T and B cells in the context of non-natural immune attenuation. We speculate that long COVID may have different immune phenotypes, and therefore requires individualized treatment approaches.

One notable advantage of our study is that our cohort has a clear immune background, being comprised exclusively of patients who were infected with the original strain of SARS-CoV-2 during the first wave of the pandemic and have not undergone vaccination or re-infection. This is significant as the extent to which these factors contribute to the risk of developing long COVID in COVID-19 patients is not yet fully understood.

Our study has certain limitations, such as the small sample size of participants undergoing cellular immunity analysis, which may restrict the broad applicability of our results. Additionally, we did not perform a thorough analysis of circulating follicular helper T cells and B cells. Future studies should collect samples longitudinally from multiple time points and conduct more comprehensive analyses to elucidate the underlying mechanisms of long COVID.

To sum up, this study showed that patients with long COVID had higher levels of RBD-IgG antibodies than those who fully recovered. However, we did not find any evidence of strong immune cell responses against the virus. As there may be multiple potential causes of long COVID, it is imperative to avoid adopting a “one-size-fits-all” approach to future treatment modalities.

## Supplementary Data

Supplementary materials are available at *Open Forum Infectious Diseases* online. Consisting of data provided by the authors to benefit the reader, the posted materials are not copyedited and are the sole responsibility of the authors, so questions or comments should be addressed to the corresponding author.

## Supplementary Material

ofae137_Supplementary_Data
