# Supplementary material for: Immune Responses in Discharged COVID-19 Patients With and Without Long COVID Symptoms
Source: Open Forum Infect Dis. 2024 Apr 1;11(4):ofae137. doi: 10.1093/ofid/ofae137 (PMC10993057; doi:10.1093/ofid/ofae137)
Supplement: ofae137_Supplementary_Data [file ofae137_supplementary_data.docx]

**Immune Responses in Discharged COVID-19 Patients with and without Long COVID Symptoms**

**Inclusion and exclusion criteria:**

This study was done at Jin Yin-tan Hospital, the first designated hospital for patients with COVID-19 in Wuhan, Hubei, China. We included all patients with laboratory confirmed COVID-19 who were discharged from Jin Yin-tan Hospital between Jan 7, and May 29, 2020. We excluded the following patients: (1) those who died before the follow-up visit, (2) those for whom follow-up would be difficult owing to psychotic disorder, dementia, or re-admission to hospital attributed to underlying diseases, (3) those who were unable to move freely due to concomitant osteoarthropathy or immobile before or after discharge due to diseases such as stroke or pulmonary embolism, (4) those who declined to participate, (5) those unable to be contacted, and (6) those living outside of Wuhan or in nursing or welfare homes. All discharged patients met uniform discharge criteria according to the Chinese clinical guidance for COVID-19 pneumonia diagnosis and treatment issued by the National Health Commission (ie, no fever for 3 consecutive days, improvement in respiratory symptoms, obvious resolution and recovery of acute lesion in lung imaging, and two negative test results for SARS-CoV-2 24 h apart).

**Immunological evaluation methods:**

**Plasma and PMBC isolation**

Venous blood was collected from participants and processed within 12 h to isolate plasma and peripheral blood mononuclear cell (PBMCs). Plasma was separated by centrifugation at 300 xg for 10 minutes and stored at -80°C until testing. PBMCs were isolated from blood using Ficoll-Paque PLUS (GE Healthcare, Chicago, IL) according to the manufacturer’s instructions. Isolated PBMCs were frozen in 90% heat- inactivated fetal bovine serum (FBS, Hyclone, Northbrook, IL) supplemented with 10% DMSO (Sigma- Aldrich, St. Louis, MO, USA), and stored in liquid nitrogen before analysis.

**Enzyme-linked immunosorbent assay (ELISA)**

Titres of IgA, IgM, and IgG antibodies against the N, S, and RBD of SARS-CoV-2 were evaluated using the enzyme-linked immunosorbent assay (ELISA). Briefly, 20 ng of N, S, and RBD protein (Sino Biological, Beijing, China) were used as coating protein, respectively. Plasma samples were diluted 1/400 with 0·5% bovine serum albumin (BSA) and incubated for 1 h at 37°C. After washing, horseradish peroxidaseconjugated goat anti-human Fc5μ fragment specific polyclonal IgM (Jackson ImmunoResearch, West Grove, PA, USA), rabbit anti-human α chain specific polyclonal IgA (Jackson ImmunoResearch), and goat anti-human Fc specific polyclonal IgG (Sigma Aldrich, St Louis, MO, USA) antibodies were added to the plates at a dilution of 1/60 000 with 0·5% BSA. After 1 h of incubation at 37°C, the plates were washed and developed with 100 μL substrate solutions A (3,3’,5,5’- tetramethylbenzidine) and B (hydrogen peroxide) in each well (Wantai Biotech Corp, Beijing, China). The reaction was stopped by adding 50 μL of 2 M sulfuric acid. Optical density at 450 nm (OD450) was determined with a multifunctional microplate reader SpectraMax M5 (Molecular Devices, Sunnyvale, CA, USA). Cut-off values were determined by calculating the mean absorbance at 450 nm of negative plasma plus 3-fold SD values, which were 0·30, 0·24, 0·20 for IgM, 0·2, 0·26, and 0·2 for IgA, 0·2, 0·2, and 0·2 for IgG against SARS-CoV-2 N, S and RBD respectively.

**Microneutralization assay**

NAbs were assessed on Vero cells (ATCC, Manassas, VA, CCL-81) infected with SARS-CoV-2 (IPBCAMS-WH-01/2019, no. EPI_ISL_402123, Wuhan strain hereafter), D614G, Beta, and Delta variants (all these strains were isolated from COVID-19 patient respiratory tract samples in biosafety level 3 laboratory by Institute of Pathogen Biology, Chinese Academy of Medical Sciences) using a microneutralization assay. A serial two-fold dilution of serum samples (starting at 1:10) was preincubated with SARS-CoV-2 at 100 50% tissue culture infective doses for 2 h at 37°C, and the virus-serum mixture was added to Vero cells and incubated for 1 h. The cytopathic effect was assessed 5 days after incubation. Four duplicate wells were used for each serum dilution. Neutralising antibody titres were calculated using the Reed-Muench method. Viral back-titration was done, and serum samples known to be positive for neutralising antibodies were used as a positive control in each test. The cut-off for a positive NAb titre was 1/10.

**Peptide synthesis**

A total of 347 15- to 18-mer peptides that overlap by 10 amino acid residues and span the S, N, membrane (M), envelope (E), ORF3a, ORF6, ORF7a, and ORF8 proteins of SARS-CoV-2,1 and a total of 158 18- mer peptides that overlap by 10 amino acid residues targeting S protein of Beta variant were synthesized (purity >90%; Sangon Biotech, Shanghai, China). The pool contained cytomegalovirus (CMV), Epstein- Barr virus (EBV), and Influenza viruses (IFV) specific epitopes (CEF peptide pool) was used as a control.

**Ex-vivo ELISpot assay**

Ex-vivo Interferon-γ (IFN-γ) enzyme-linked immunospot (ELISpot) assays were performed using cryopreserved PBMCs with a Human IFN-γ ELISpot kit (Mabtech, NS, Sweden) following the manufacturer’s instructions. Briefly, cryopreserved PBMCs were thawed and washed once with the RPMI-1640 medium containing 10% FBS and rested overnight prior to assay. Human IFN-γ pre-coated plates were washed with phosphate-buffered saline (PBS), and blocked with culture media containing 10% FBS for 30 min. Total 2×105 PBMC per well were stimulated in duplicates with overlapping peptide pools at a final concentration of 2 μg/ml for 24 h. DMSO were used as negative controls. Phorbol myristate acetate (PMA)/ionomycin (Multi Science, Hangzhou, China) was used as positive control. Spots were counted using an AID ELISPOT Reader System (AID GmbH, Strasberg, Germany). Mean spots of the negative control wells were subtracted from the test wells to quantify the intensity of antigen- specific T cell responses, and the results were presented as Spot Forming Unit per 106 PBMC (s.f.u./106 PBMC). T cell responses were considered positive if the mean spot count was ≥ 3-fold higher than the mean spot of the negative control and ≥20 s.f.u./106 PBMCs. If negative control wells had >100 s.f.u. 106 PBMC or positive control wells had <1000 s.f.u/106 PBMC, the results were excluded from further analysis.

**PBMC in *vitro* expansion culture**

For in vitro culture, 1×106 PBMCs were plated in 24-well plates and pulsed as a pellet for 1 h at 37 °C with 10 μM of SARS-CoV-2 peptides and cultured with fresh PRMI 1640 (Gibco, Thermo Fisher Scientific, Massachusetts, US) supplemented with 10% human AB serum and 1% penicillin/streptomycin (Sigma-Aldrich, St. Louis, MO), under addition of 100 U/ml recombinant human interleukin (rIL-2, PeproTech, Rocky Hill, NJ). During culturing, half of the medium was changed on days 3, 5, and 7. The cells were subcultured when needed. Expanded PBMCs were analyzed by ELISpot assay and intracellular cytokine staining (ICS).

**ELISpot assay using expanded PBMCs**

Expanded PBMCs were washed once with the RPMI-1640 medium containing 10% FBS and rested overnight prior to assay. IFN-γ ELISpot assays were performed using a Human IFN-γ ELISpot kit (Mabtech, NS, Sweden) following the manufacturer’s instructions, as ex-vivo ELISpot assay.

**Intracellular cytokine staining (ICS)**

PBMCs were incubated with pooled peptides at a final concentration of 10 μg/mL in the presence of 1 μg/ml monoclonal antibodies CD28 and CD49d for 1 h, and then with brefeldin A (GolgiPlug, Biolegend, San Diego, CA) and monensin (GolgiStop, Biolegend) for an additional 5 h. Dead cells were labeled using BD Horizon Fixable Viability Stain 510 (BD Biosciences). Surface staining was performed using PerCP-Cy5.5-anti-CD3, BV650-anti-human CD4, PE-Cy7-anti-human CD8 (Biolegend). After incubation with fixation permeabilization stain buffer (Invitrogen, Carlsbad, CA), cells were immunostained using BV421-anti-IFNγ, BV711-anti-TNFα, APC-anti-IL-2 (Biolegend). Information about reagents used for intracellular cytokine staining (ICS) are listed in appendix pp 9–10. No peptide stimulation was used as a negative control for each condition. Specific cytokine responses were calculated by subtracting the background activation before further analysis. T cells exposed to PMA/ionomycin served as positive controls. All samples were acquired on a BD LSRFortessa (BD Biosciences) flow cytometer and analyzed using FCS Express 7 (De NOVO software, Pasadena, CA). Single-stained CompBeads (BD Biosciences) or single-stained PBMCs were used for compensation. Unstained PBMCs were used for assessing autofluorescence.

**Table S1. Characteristics of the enrolled COIVD-19 patients**

|  | **Total (n=1041)** | **CC (n=561)** | **LC (n=480)** | ***P*** |
| --- | --- | --- | --- | --- |
| **Age, years** | 57.6±12.5 | 57.1±13.2 | 58.2±11.7 | 0.146 |
| **Sex** |  |  |  | **0.020** |
| Men | 561 (53.9%) | 321 (57.2%) | 240 (50.0%) |  |
| Women | 480 (46.1%) | 240 (42.8%) | 240 (50.0%) |  |
| **Comorbidities** | 408 (40.1%) | 223 (40.5%) | 185 (39.6%) | 0.763 |
| Hypertension | 293 (28.8%) | 164 (29.8%) | 129 (27.6%) | 0.479 |
| Diabetes | 128 (12.6%) | 76 (13.8%) | 52 (11.1%) | 0.139 |
| Cardiovascular diseases | 66 (6.5%) | 22 (4.0%) | 44 (9.4%) | **0.001** |
| COPD | 16 (1.6%) | 9 (1.6%) | 7 (1.5%) | 0.861 |
| Chronic kidney disease | 9 (0.9%) | 5 (0.9%) | 4 (0.9%) | 0.929 |
| **Illness severity** |  |  |  | 0.633 |
| 3 | 266 (26.2%) | 146 (26.5%) | 120 (25.7%) |  |
| 4 | 685 (67.4%) | 372 (67.6%) | 313 (67.0%) |  |
| 5-6 | 66 (6.5%) | 32 (5.8%) | 34 (7.3%) |  |
| **Time from symptom onset to follow-up, days** | 349.0 (337.0-361.0) | 349.0 (337.0-361.0) | 350.0 (342.0-362.0) | 0.573 |

**Table S2. Characteristics of the enrolled patients** **for assessing SARS-CoV-2-specific memory T cells**

|  | **Total (n=75)** | **CC (n=40)** | **LC (n=35)** | ***P*** |
| --- | --- | --- | --- | --- |
| **Age, years** | 57.6±11.9 | 56.9±12.8 | 58.3±11.0 | 0.598 |
| **Sex (Man/Women)** | 48/27 | 24/16 | 24/11 | 0.440 |
| **Comorbidity** | 28 (38.9%) | 17 (42.5%) | 11 (34.4%) | 0.482 |
| Hypertension | 18 (25%) | 14 (35%) | 4 (12.5%) | 0.028 |
| Diabetes | 6 (8.3%) | 3 (7.5%) | 3 (9.4%) | 0.775 |
| Cardiovascular diseases | 10 (13.9%) | 5 (12.5%) | 5 (15.6%) | 0.703 |
| COPD | 2 (2.8%) | 0 (0%) | 2 (6.3%) | 0.109 |
| Chronic kidney disease | 0 (0%) | 0 (0%) | 0 (0%) | NA |
| **Illness severity** |  |  |  | 0.173 |
| 3 | 29 (40.3%) | 13 (32.5%) | 16 (50%) |  |
| 4 | 24 (29.2%) | 15 (37.5%) | 9 (25.7%) |  |
| 5-6 | 22 (30.6%) | 12 (30%) | 10 (31.3%) |  |


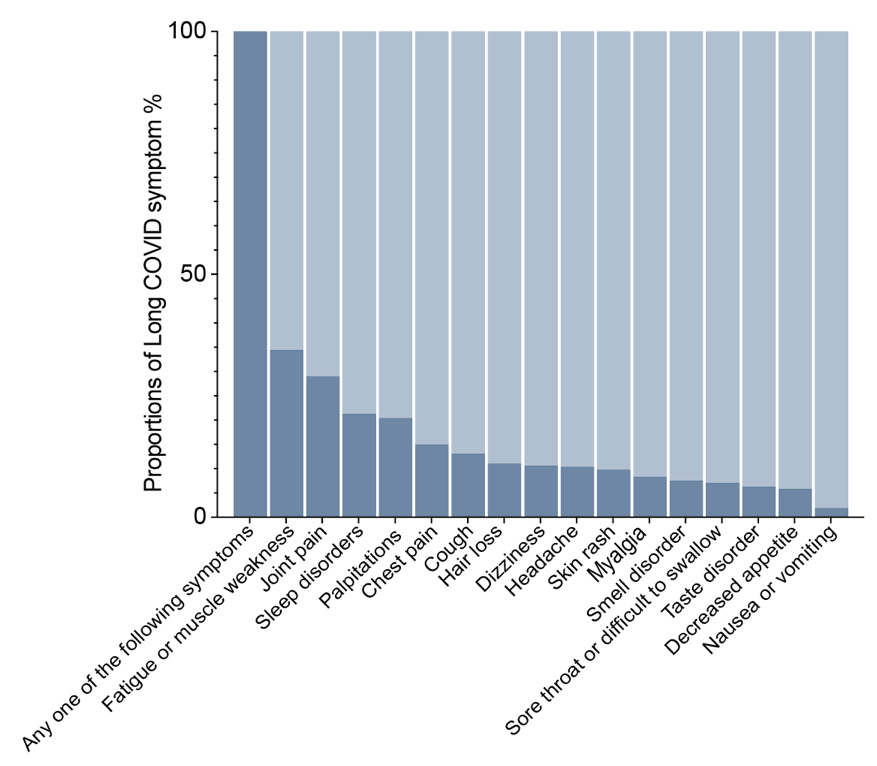


**Figure S1. Proportions of symptoms reported by Long COVID patients**


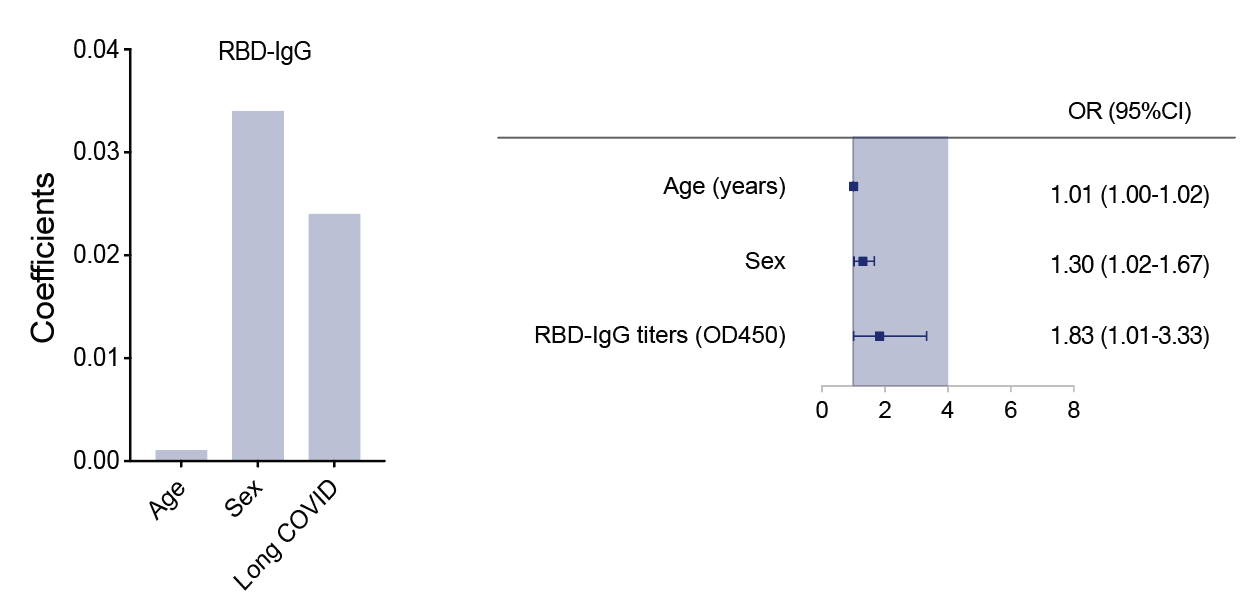


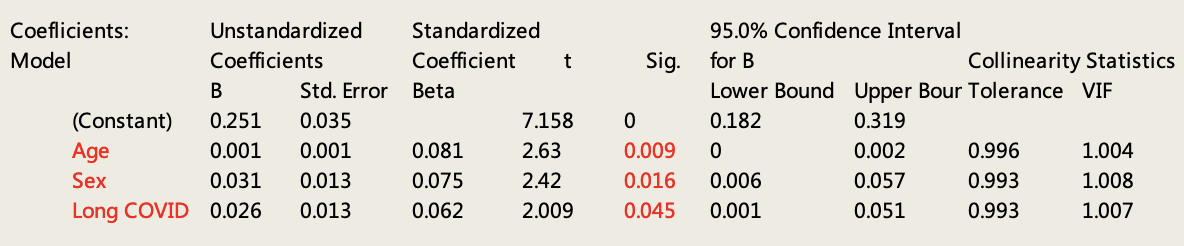


**Figure S2. Model coefficients from Multiple Linear Regression analysis**

**
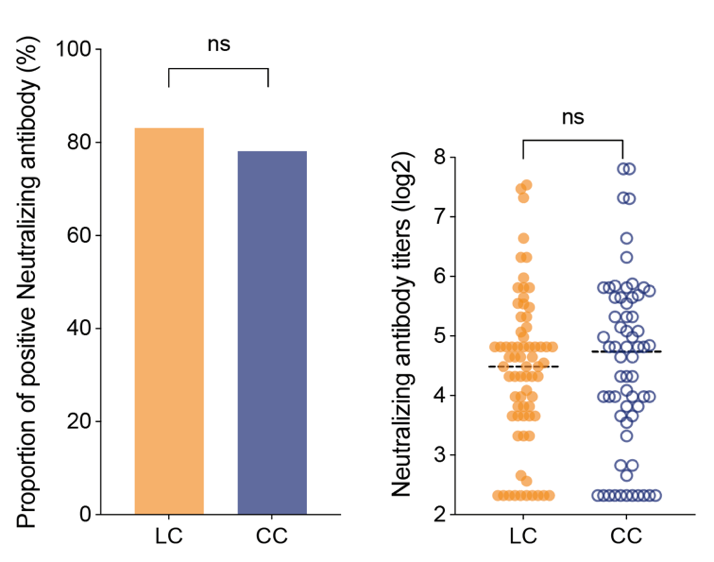
**

**Figure S3. Microneutralisation assays results of LC and CC patients**


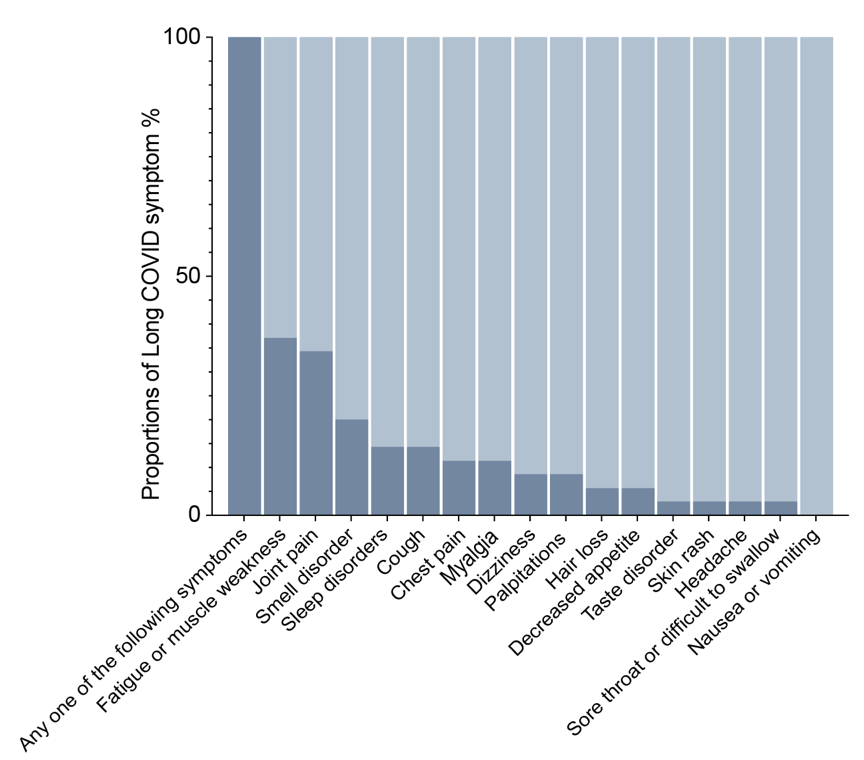


**Figure S4 Proportions of self-reported sequelae symptoms in patients with LC in the cellular immune part**


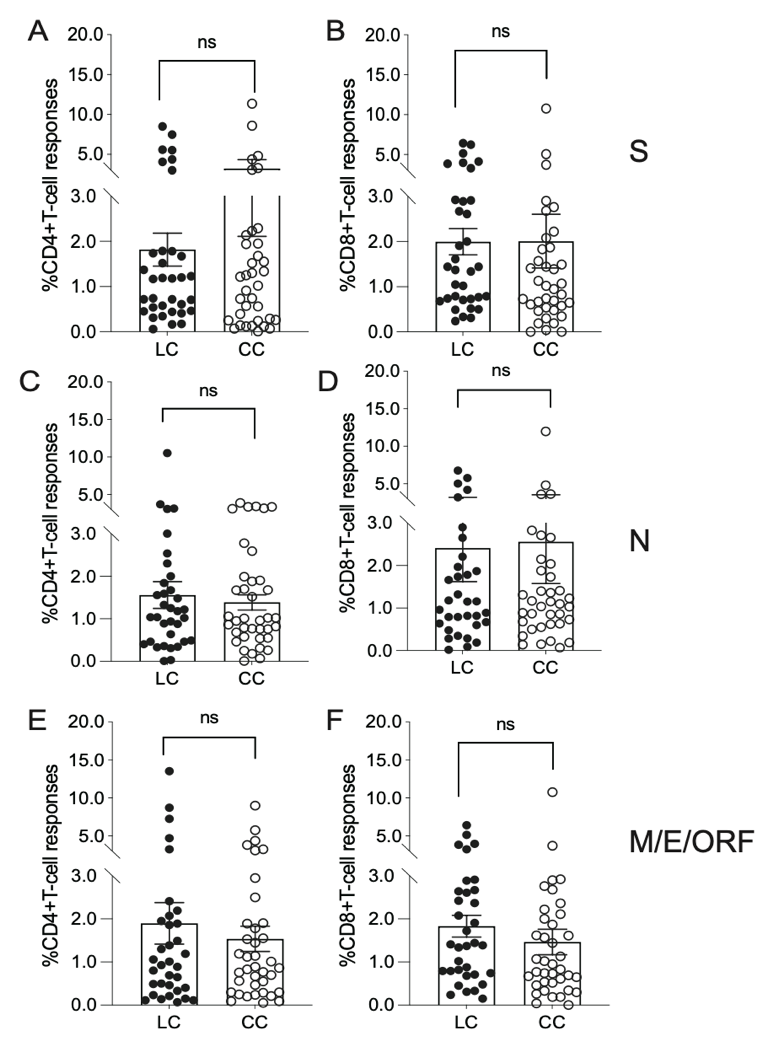


**Figure S5 Quantitative Evaluation of cytokine producing Spike-, N-, and M/E/ORF-specific memory CD4+ and CD8+ T cells**

Note: (A-B) Proportion of responded Spike-specific CD4+ (A) and CD8+ (B) T cells in LC and CC patients; (C-D) Proportion of responded N-specific CD4+ (C) and CD8+ (D) T cells in LC and CC patients; (E-F) Proportion of responded M/E/ORF-specific CD4+ (E) and CD8+ (F) T cells in LC and CC patients.
